# Biological Effects of Small Sized Graphene Oxide Nanosheets on Human Leukocytes

**DOI:** 10.3390/biomedicines12020256

**Published:** 2024-01-23

**Authors:** Michele Aventaggiato, Federica Valentini, Daniela Caissutti, Michela Relucenti, Marco Tafani, Roberta Misasi, Alessandra Zicari, Sara Di Martino, Sara Virtuoso, Anna Neri, Stefania Mardente

**Affiliations:** 1 Department of Experimental Medicine, Sapienza University, Viale Regina Elena, 00161 Rome, Italy; michele.aventaggiato@uniroma1.it (M.A.); daniela.caissutti@uniroma1.it (D.C.); marco.tafani@uniroma1.it (M.T.); roberta.misasi@uniroma1.it (R.M.); alessandra.zicari@uniroma1.it (A.Z.); saradimartino01@universitadipavia.it (S.D.M.); 2 Department of Sciences and Chemical Technologies, Tor Vergata University, Via della Ricerca Scientifica 1, 00133 Rome, Italy; federica.valentini@uniroma2.it; 3 Department of Anatomical, Histological, Forensic and Orthopaedic Sciences, Sapienza University of Rome, Via Alfonso Borelli 50, 00161 Rome, Italy; michela.relucenti@uniroma1.it; 4 Higher Institute of Health (ISS), Viale Regina Elena 299, 00161 Rome, Italy; sara.virtuoso@iss.it; 5 Department of Biomedicine and Prevention, Tor Vergata University, Viale Montpellier, 1, 00133 Rome, Italy; anna.neri@uniroma2.it

**Keywords:** graphene oxide, nanosheets, monocytes, macrophages, chemotaxis, reactive oxygen species, tumour antigens

## Abstract

Since the discovery of graphene, there has been a wide range of the literature dealing with its versatile structure and easy binding of biomolecules as well as its large loading capacity. In the emerging field of immunotherapy, graphene and its derivatives have potential uses as drug delivery platforms directly into tumour sites or as adjuvants in cancer vaccines, as they are internalized by monocytes which in turn may activate adaptive anti-tumoral immune responses. In this study, we expose cells of the innate immune system and a human acute monocytic leukemia cell line (THP-1) to low doses of small-sized GO nanosheets functionalized with bovine serum albumin (BSA) and fluorescein isothiocyanate (FITC), to study their acute response after internalization. We show by flow cytometry, uptake in cells of GO-BSA-FITC reaches 80% and cell viability and ROS production are both unaffected by exposure to nanoparticles. On the contrary, GO-BSA nanosheets seem to have an inhibitory effect on ROS production, probably due to their antioxidant properties. We also provided results on chemotaxis of macrophages derived from peripheral blood monocytes treated with GO-BSA. In conclusion, we showed the size of nanosheets, the concentration used and the degree of functionalization were important factors for biocompatibility of GO in immune cells. Its low cytotoxicity and high adaptability to the cells of the innate immune system make it a good candidate for deployment in immunotherapy, in particular for delivering protein antigens to monocytes which activate adaptive immunity.

## 1. Introduction

In recent years, scientific communities have made numerous efforts to evaluate the biocompatibility of different types of nanomaterials in the emerging fields of nanomedicine and nanopharmacology [1]; for each of these materials, short- and long-term studies on cytotoxicity in relation to biological systems were needed [2]. The ideal nanomaterial for drug delivery applications should have a large surface area, an ability to bind different biomolecules, stability in biological fluids [3], and a capacity to penetrate solid tissues and/or exert anti-microbial properties [4].

Graphene (G) is a promising nanomaterial for biomedical applications because of its unique atomic structure, which allows functionalization, and its high surface area-to-volume ratio, as well as a relevant loading capacity of bioactive molecules, genes and miRNA for drug/miRNA delivery, and gene transfection purposes [5]. Synthesis methods of G and graphene oxide (GO) derivatives determine the size, solubility, and biocompatibility [6,7,8]. One particularly interesting application of GO nanoparticles in cancer is drug delivery into tumour sites [9,10] and the possibility of using them as adjuvants in immune therapy, by facilitating the delivery and availability of tumour antigens to antigen-presenting cells (APCs) to induce strong and durable adaptive immune responses against tumours. Since activation of adaptive immune response is strictly related to antigen presentation by innate immune cells such as dendritic cells, monocytes, and macrophages, the use of nanoparticles that target APCs in anticancer immunotherapy can lead to the development of personalized therapeutic strategies or cancer vaccination. Studies of biocompatibility and cytotoxic effects of graphene-based nanomaterials, in cancer or on the immune system, show there are differences related to the way graphene is synthesized, to the physical form being given to the nanoparticle, to the oxidation state (as GO tends to be less toxic than its reduced forms) or to the concentration of the nanoparticle [11]. Furthermore, the functionalization of GO plays a crucial role in determining the cytotoxic effects on cells [12]. The effects exerted on cells of the immune system have been studied in the short-term in a number of ways, namely the metabolizing and biodegrading activity of GO in immune cells [13,14], the possibility of using short-term primary cultures without the need for immortalizing agents and the quick response-resolution of effects like the release of acute phase cytokines and reactive oxygen species (ROS) [15], typical of innate immune cells such as monocytes [16]. These kinds of acute cytotoxic effects can lead to DNA mutations and genotoxicity [17]. 

The present study focused on the biocompatibility of GO functionalized with bovine serum albumin (BSA) and fluorescein isothiocyanate (FITC) in healthy human leukocytes and in a human acute monocytic leukemia cell line (THP-1). Primary culture of healthy monocytes and their derived macrophages, as well as a long-term cell line, were exposed to low doses of GO-BSA-FITC and their uptake, growth, ROS production, and chemotactic ability were analyzed.

## 2. Materials and Methods

### 2.1. Materials

Electrochemically grown GO was obtained by synthetic approach as previously reported [18,19]. BSA protein and fluorescein isothiocyanate (FITC), were purchased from Sigma–Aldrich (Merck, Darmstadt, Germany). All reagents were of analytical grade and used as received. A Milli-Q pore apparatus system was applied to produce distilled water for all aqueous solutions.

### 2.2. Atomic Force Microscopy (AFM)

AFM measurements were performed in air using a Park XE-120 microscope (Park Systems, Suwon, Republic of Korea). Experiments were carried out in tapping mode by using Si tips with a spring constant of about 40 N/m and a typical curvature radius on the tip of 7 nm. GO powder was dispersed in distilled water at final concentration of 1 mg/mL by applying a polytronic probe (Kinematica™ Polytron™ 1200E, 1300D and 2500E Generator/Probes, Malters, Switzerland), for 30 min. A few droplets of nanodispersions were then deposited on mica substrates.

### 2.3. UV−Vis and Fluorescence Spectrometry

UV−Vis spectra and fluorescence spectra were collected with Shimadzu UV-2550 spectrophotometer (Tokyo, Japan) and Hitachi F-4600 fluorescence spectrometer (Tokyo, Japan) respectively.

### 2.4. Zeta Potential and Polydispersity Index (PDI)

The zeta potential measurements and PDI and were carried out using Zetasizer Nano ZS equipment (Malvern, UK). This apparatus is equipped with a back scattering detection mode (with an angle of 173), and a laser He-Ne with wavelength at 633 nm.

### 2.5. Synthesis of GO, GO-BSA and GO-BSA-FITC (Fluorescent Dye Labeling of BSA Protein)

**Scheme 1:**
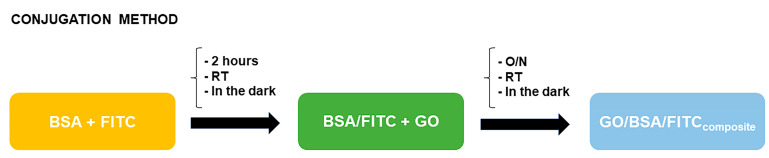
The conjugation method.

GO was synthesized as described in our previous papers [18,19]. An aqueous solution of BSA (10 mg/mL) was gently mixed with an excess FITC, previously dispersed in DMSO (dimethyl sulfoxide). Both reagents were allowed to react for 2 h at room temperature in the dark. The resulting FITC-labeled BSA protein (i.e., BSA-FITC composite) was purified by dialysis to eliminate the excess of non-adsorbed FITC. The concentration of the resulting BSA-FITC composite material after dialysis was quantified by bicinchoninic acid (BCA) protein assay test. The conjugation method, illustrated in the flow chart below (Scheme 1), was described in [20].

BSA Protein loading onto GO nanosheets was obtained by mixing BSA-FITC with GO dispersed in double distilled water (0.25 mg/mL) at room temperature overnight in darkness. Unspecifically adsorbed and unbound BSA-FITC was removed by successive washings and centrifugations as previously reported [21].

### 2.6. Cell Culture

The THP-1 cell line (ECACC, Salisbury, UK) was an immortalized, human monocytic leukemia derived cell line grown in RPMI 1640 medium (Sigma Aldrich-MERCK) supplemented with 10% FBS (Sigma-Aldrich, St. Louis, MO, USA), 2 mMol/L-glutamine (Sigma–Aldrich–MERCK, St. Louis, MO, USA), 100 units/mL penicillin, and 0.1 mg/mL streptomycin (Sigma–Aldrich–MERCK, St. Louis, MO, USA) and kept at 37 °C, 5% CO_2_ in a humidified tissue culture incubator.

### 2.7. Cell Viability Assay

Primary cultures and THP-1 cells (1 × 10^5^ cells/mL) were seeded in 96-well microplates for 24 h. After 24 h, the medium was replaced with medium containing 2% FBS and GO-BSA or GO-BSA-FITC for 3 h or 24 h at two different concentrations (1.5 μg/mL or 3 μg/mL). Negative controls consisted of cells resuspended in RPMI supplemented with 2% FBS. After an additional 24 h of culture, cell viability was measured using the water-soluble tetrazolium-1 assay (WST) (Abcam, Cambridge, UK) according to the manufacturer’s instructions. The optical density was measured using a microplate reader (DAS, Rome, Italy).

### 2.8. Leukocyte Isolation from Peripheral Blood

Blood was obtained from male healthy donors (aged from 20 to 40) after written informed consent. Peripheral blood mononuclear cells (PBMCs) were isolated by Histopaque 1119 density gradient centrifugation (Sigma–Aldrich, USA). Monocytes were then purified using anti-CD14 monoclonal antibodies conjugated to magnetic beads (Miltenyi Biotec, Bergisch Gladbach, Germany) according to the manufacturer’s instructions. The purified monocytes were then cultured in RPMI 1640 supplemented with 10% fetal bovine serum (FBS), 2 mM L-Glutamine, and 100 IU/mL penicillin-streptomycin (complete medium), seeded on a coverslip in 6-well plates (5 × 10^5^ cells/well) or in the 24-well transwell System (Thermofisher, Waltham, MA, USA) (3 × 10^5^ cells/well) and cultured for 5–6 days at 37 °C with 5% CO_2_ in the presence of 33 ng/mL Granulocyte Macrophage-Colony Stimulating Factor (GM-CSF, R&D System, Minneapolis, MN, USA) to obtain type 1 oriented macrophages, whose phenotype was confirmed by flow cytometry. Cells were used for subsequent GO treatments and chemotaxis experiments described below. Neutrophils [22,23] were separated from mononuclear ring by pipetting the interface between pellets containing red blood cells and mononuclear rings and placing them into a Histopaque-1077 gradient (Sigma–Aldrich, USA).

Established cultures of neutrophils, lymphocytes, and monocytes were kept at 37 °C for 24 h before starting the treatment with either GO-BSA or GO-BSA-FITC for 3 h or 24 h at two different concentrations (1.5 μg/mL or 3 μg/mL).

### 2.9. Flow Cytometry

Immunostaining with primary antibodies—anti CD11-PE (BD Biosciences, Oxford, UK) for neutrophils, anti CD3-PE (BD Biosciences) for lymphocytes, anti CD14-PE (BD Biosciences) for monocytes—was used for gating the different leukocyte subsets treated or untreated with GO-BSA-FITC. Each antibody was diluted 1:100 and added to cellular suspensions for 30 min in the dark at room temperature. At the end of incubation time, samples were washed with PBS and analyzed by flow cytometry (Cytoflex, Beckman-Coulter, Oxford, UK).

### 2.10. Scanning Electron Microscopy

Macrophages were grown on glass disks and processed as reported in [24,25,26,27], as follows: fixation fluid was glutaraldehyde 2.5% in PBS pH 7.4 at 4 °C for at least 48 h; washing solution used was PBS pH 7.4 20 min × 2 times; post-fixation solution was OsO_4_ 1.33% in H_2_O 1 h. Following washing in PBS pH 7.4 20 min × 2 times, dehydration was performed in ascending ethanol series (30%, 50%, 70%, 96% 10 min each, 100% 10 min × 3). The substitution procedure used was: HDMS 50% ethanol 50% 10 min, HDMS 100% 10 min. The samples were then mounted on aluminum stubs with carbon tape and sputter coated with platinum (2 min 15 mA × 2 times, sputter coater Emitech K500X, Emitech Groupe, Montigny le Bretonneux, France). Samples were observed under a Hitachi SU 3500 scanning electron microscope (Hitachi, Tokyo, Japan) at operating conditions of high vacuum and 8–10 kV.

### 2.11. Confocal Microscopy

Macrophages were treated with GO-BSA-FITC and, at the same time, labeled with CellTrace™ Calcein Red-Orange (ThermoFisher C34851, Waltham, MA, USA) at the final concentration of 500 nM for 30 min. After treatment, cells were washed three times with PBS, spotted on a glass slide, and analyzed with a LSM510 confocal microscope (Zeiss, Oberkochen, Germany).

### 2.12. Reactive Oxygen Species (ROS) Detection

ROS formation in short-term cultures and in THP-1 cell line treated or untreated with nanocomposites (at indicated concentrations and times) was assayed by flow cytometry using the dye DCFH-DA (2′,7′-dichlorofluorosceine-diacetate) (Sigma–Aldrich, USA) and following standard methods [28].

After treatment of cells with 1.5 μg/mL or 3 μg/mL GO-BSA for 3 h and 24 h, DCFH-DA (2′7′ dichlorofluorescein-diacetate) (Merck) (final concentration 1 μM) was added to cell cultures for 15 min at 37 °C. After incubation, the cells were washed in PBS and analyzed using a flow cytometer (Cytoflex, Beckman Coulter, Brea, CA, USA) equipped with an Argon laser at 488 nm. Cells were gated on the basis of forward angle light-scatter (FSC) and 90 light scatter (SSC) parameters. For every histogram, a minimum of 20,000 events were counted. Mean fluorescence intensity was detected and expressed as a percentage of relative ROS level versus control cells. 

### 2.13. Chemotaxis Assay

For chemotaxis assays, transwell migration chambers were used according to the manufacturer’s protocols (Corning Incorporated, New York, NY, USA). Briefly a suspension of 10^6^ cells was added to the upper chamber of the transwells and allowed to settle for 2 h. The transwells were then moved to the wells containing GO-BSA (1.5 μg/mL or 3 μg/mL) and the substances used as positive or negative controls, i.e., LPS (L4391 Sigma–Aldrich) and necrotic extracts (10 ng/mL) obtained from freezing and thawing pellets of the anaplastic thyroid cell line CAL-62 (5 × 10^6^ cells). The transwell filters were then stained with 0.5% crystal violet (Merck; C0775) and analyzed using Zeiss IM35 microscope (Zeiss) and a digital camera (Nikon Digital Sight DSL1, Tokyo, Japan). Finally, according to the manufacturer’s instructions, stained cells were dissolved with 10% acetic acid (100 µL/well) for colorimetric reading of OD at 560 nm with Glomax^®^-Multi Detection System (Promega, Milan, Italy) [29].

### 2.14. Statistics

Statistical analysis was carried out using KaleidaGraph version 4.5.1 (Synergy Software Inc., Reading, PA, USA). Data are expressed as means ± SD. *p* < 0.05 was considered a statistically significant difference. 

## 3. Results

### 3.1. Synthesis and Characterization of GO-BSA Functionalized with FITC

**Figure 1:**
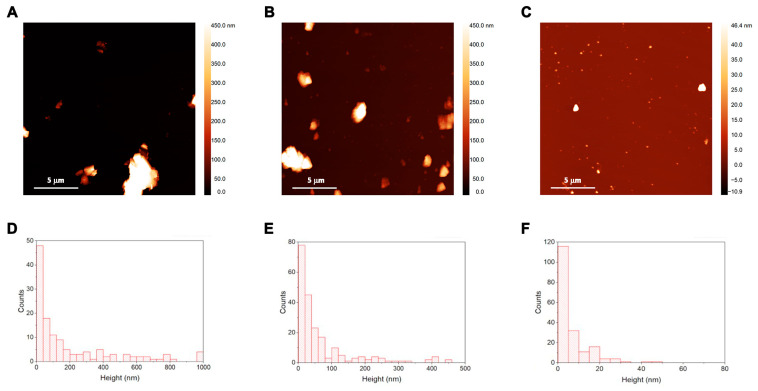
Morphology and sizes of GO and composites. AFM images of GO before (**A**) and after coating with BSA (**B**) and FITC (**C**). Histograms of the heights of GO sheets on GO sample (**D**), after coating with BSA (**E**) and with FITC (**F**).

Morphology of GO before (Figure 1) and after loading BSA-FITC was studied by Atomic Force Microscopy (AFM). GO AFM images showed a distribution of sheets with different sizes (Figure 1A), with an exponentially decreasing trend of sheet heights (Figure 1D) and tails extending to several hundred nm. The median size of the measurements is approximately 85 nm. Figure 1B shows a composite GO-BSA with a similar morphology to untreated GO, but with lower median thickness (Figure 1E). Distribution of heights shows the same decreasing trend, with a median size of around 32 nm. Samples treated with FITC chromophore (Figure 1C) showed a significant decrease in size of final composite. Height profiles show a Poisson type (Figure 1F) exponential distribution with median thickness of 3.2 nm. Lateral dimensions (Table 1) of nanosheets showed a decrease, though not as intense as for the heights, due to the chemical exfoliation procedure used during GO synthesis and the subsequent decoration/conjugation with BSA and FITC. 

Morphology and thickness of GO sheets investigated by AFM suggested successful formation of the composites GO-BSA-FITC. Final concentration of composite GO-BSA-FITC (determined by BCA protein assay test) was 20 μg/mL.

**Figure 2:**
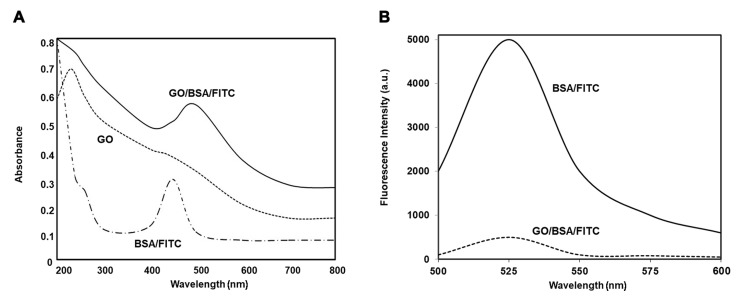
(**A**) UV−vis and (**B**) fluorescence spectra of GO before and after loading with BSA-FITC.

GO-BSA-FITC samples were also analyzed by UV−Vis and fluorescence spectroscopy, to demonstrate the success of the conjugation. UV−Vis spectra (Figure 2A) showed two absorption peaks, centered approximately at ~450 and ~220 nm for BSA-FITC and unconjugated GO respectively, while a single peak appeared centered around 495 nm for GO/BSA-FITC composite. The shift of the latter peak towards BSA-FITC absorption wavelength (~495 nm) demonstrates the successful conjugation of BSA protein and FITC chromophore on GO nanosheets, mainly due to π−π stacking and other weak intermolecular interactions/forces [30].

After loading of BSA-FITC onto GO nanosheets, fluorescence was quenched (Figure 2B), meaning there was a significant and stable interaction between BSA-FITC and GO, analyzed by fluorescence spectroscopy. 

Table 2 and Supplementary Materials show the Z-potential of the different composites becomes more negative in GO-BSA and GO-BSA-FITC than in GO alone, indicating greater dispersibility of the GO-BSA and GO-BSA-FITC composites in water medium. The polydispersity index (PDI) decreases in the composites and parallels the decrease of the GO nanosheets diameters when conjugated to BSA. Both Z potential and PDI results aligned with AFM, which showed GO-BSA and GO-BSA-FITC sizes decreased when compared to GO alone (see Figure 1). The Z-potential and PDI measurements also show the binding between GO-BSA and FITC. The most negative Z potential (−42.8 mV) and lowest PDI index (0.382) values are found in the GO-BSA-FITC sample, indicating final conjugation with FITC improves dispersion in water. 

### 3.2. Uptake of GO by Different Leukocyte Populations and THP-1 Cells

**Figure 3:**
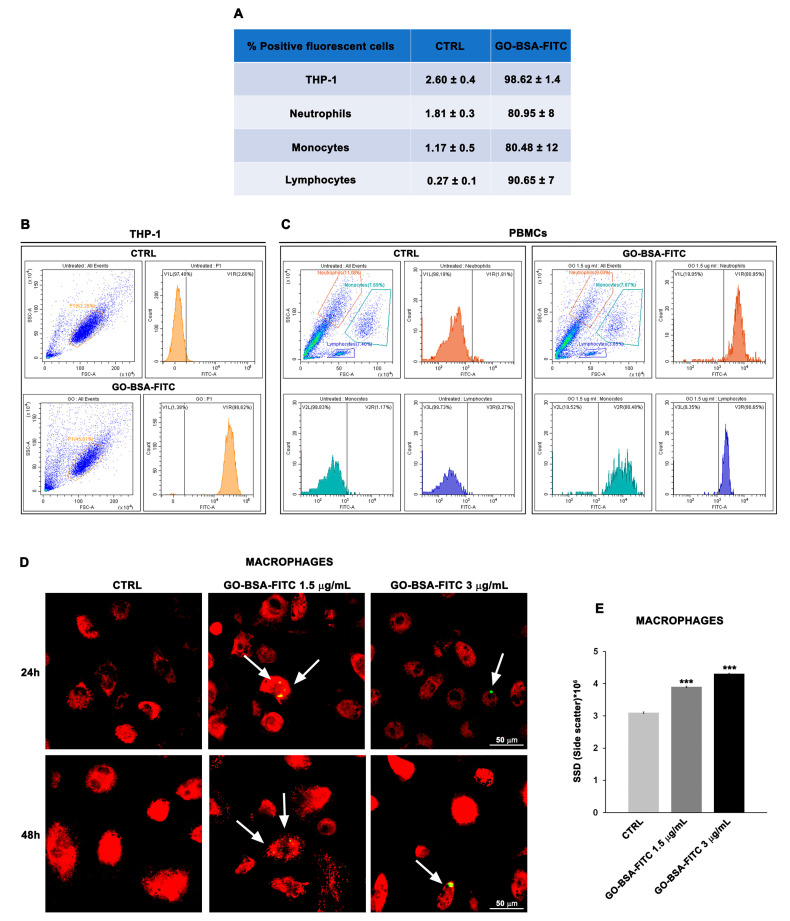
Uptake of GO-BSA-FITC in primary cultures of leukocytes, human macrophages, and THP-1 cells. (**A**) Table summarizes percentage of positive fluorescent cells, (**B**) graphs of fluorescent neutrophils, lymphocytes, and monocytes, untreated or treated with 3 μg/mL GO-BSA-FITC, (**C**) graphs of positive fluorescent THP-1 cells treated or untreated with GO-BSA, (**D**) confocal microscopy images of macrophages treated or untreated (CTRL) with different concentrations of GO-BSA-FITC for 24 h and 48 h collected at 63× magnification (scale bar = 50 µm) arrows indicate GO-fluorescence, (**E**) histograms showing the shift in side scatter from untreated macrophages (CTRL) to treated with 1.5 and 3 μg/mL GO-BSA-FITC. (***) *p* < 0.001 compared to unexposed control (CTRL).

Different concentrations of GO-BSA-FITC were added to THP-1 cells and primary cultures of peripheral blood monocytes, neutrophils, and lymphocytes 24 h after establishment of cultures (Figure 3A, table). As shown in Figure 3B, fluorescence of GO treated cells was higher than in the controls of all considered cell populations, as demonstrated by the shift of the fluorescence peaks in the graphs (Figure 3B,C).

Confocal microscopy, carried out in human macrophages treated with GO-BSA-FITC, showed internal fluorescence in intact cells (Figure 3D). Flow cytometry of macrophages treated with GO-BSA-FITC showed a concentration dependent shift in side scatter (Figure 3E), meaning an increase in cytoplasm granularity.

### 3.3. Effect of GO-BSA-on Cell Viability of Short-Term Leukocyte Cultures and Proliferation of THP-1 Cells

To assess toxicity of GO-BSA, primary cells, short-term cultures of neutrophils, lymphocytes, and monocytes were exposed to different concentrations of GO-BSA (1.5 μg/mL and 3 μg/mL) for 3 h and 24 h. And, cell viability was determined by WST assay.

**Figure 4:**
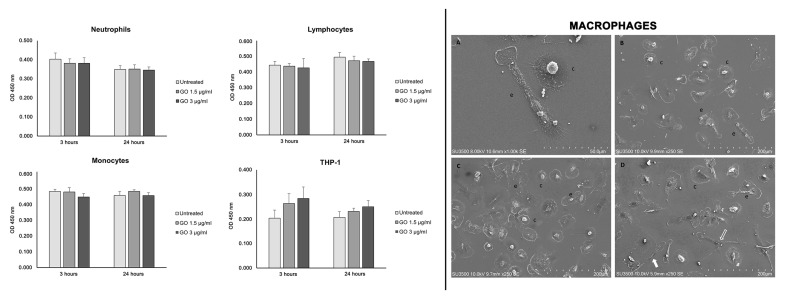
Cell viability and morphology after treatment with GO-BSA. Cell viability detected with WST at 450 nm in neutrophils, lymphocytes, and monocytes from peripheral blood and THP-1 cells, before and after treatment with GO-BSA at two different concentrations, and at 3 h and 24 h of exposure. SEM of cultured macrophages. (**A**): Control macrophages (untreated), elongated (e) and circular (c) elements 1000×, bar 50 µm. (**B**): Control macrophages with elongated (e) or circular (c) shape 250×, bar 200 µm. (**C**): Macrophages treated with GO-BSA, with elongated (e) or circular (c) shape 250×, bar 200 µm. (**D**): Macrophages treated with GO-BSA, an elongated cell with a dendritic shape was shown by an arrow; an elongated and ramified cell was shown by an empty arrow (250×, bar 200 µm).

The results suggest GO-BSA treatment does not induce changes in viability of short-term cultures leukocytes (Figure 4). GO-BSA tested in THP-1 cell line, did not induce significant changes in viability at both 3 and 24 h. It should be pointed out, although not statistically significant, the viability in THP-1 cells shows an increase that is more evident after 3 h of exposure and at the highest concentration of GO-BSA. After establishing GO-BSA was not cytotoxic either for short-term leukocyte cultures or for THP-1 cells, we investigated whether it induced morphological changes in monocyte-derived human macrophages. 

Scanning electron microscopy (Figure 4) showed macrophages adhered well to the substrate and cells were intact; cells in necrosis/apoptosis (<1 per microscopic field) were rare. Two morphological types of macrophages were observed: a circular phenotype that was established as soon as spherical cells adhered to the substrate and an elongated phenotype that was typical of cell migration (Figure 4). Circular cells adhered to the substrate and showed a prominent central nucleus, spherical or oval, and a broad, flattened cytoplasm that extended radially. The cell membrane has an irregular rim and emits numerous long filopodia and lamellipodia. Elongated macrophages also adhered well to the substrate; the nucleus could be oval with the major axis parallel to the length of the cell and protruding from the cytoplasm or slightly flattened. Generally, elongated macrophages tend to head toward surrounding cells with an amoeboid appearance, but sometimes they emit one or more filiform cell processes of considerable length and their shape may then be dendritic or branched (Figure 4B–D). The ratio between circular and elongated figures was identical in all samples (confirmed by the statistical analysis in Supplementary Materials), meaning seeded cells adhered to the substrate by taking on circular shapes and remained in the same morphological arrangement even after treatment with GO-BSA.

### 3.4. Effect of GO-BSA on ROS Production and Chemotaxis of Macrophages and THP-1 Cells

**Figure 5:**
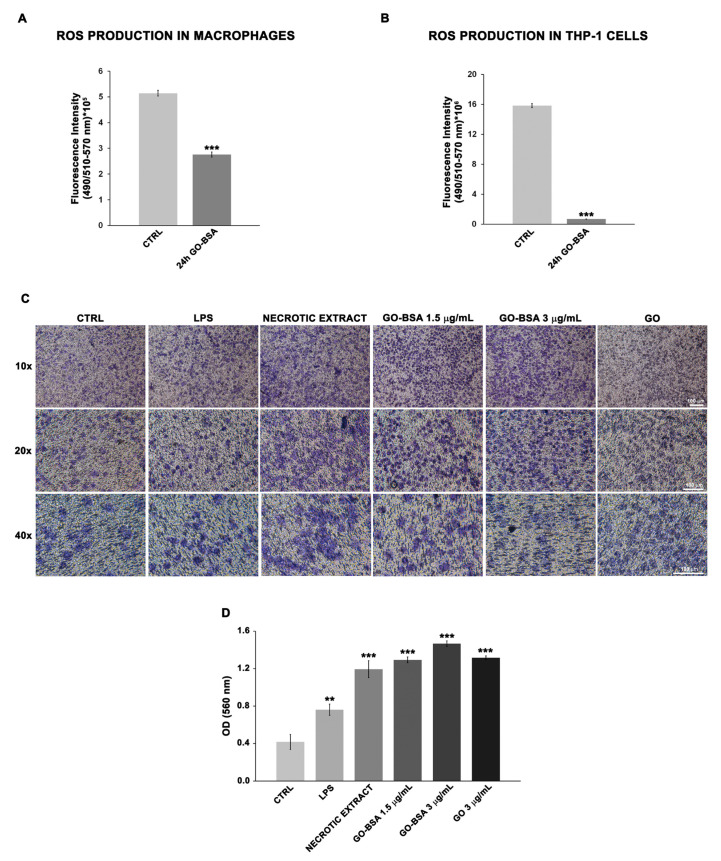
Reactive oxygen species production and chemotaxis of macrophages and THP-1 cells treated or not with GO-BSA. (**A**) Reactive oxygen species (ROS) production in macrophages and (**B**) in THP-1 cells. Histograms show mean fluorescence intensity in cells after 24 h of GO-BSA exposure. Fluorescence was detected after addition of 1 μM DCFH-DA probe for 15 min. Data are the mean ± SD of 3 different experiments. (***) *p* < 0.001 compared to unexposed control (CTRL). (**C**) Chemotaxis of macrophages against LPS, necrotic extract of tumor cells, GO-BSA (1.5 μg and 3 μg), and GO 3 μg, for 24 h (image of filters stained with crystal violet). Scale bar = 100 µm. (**D**) Histograms represent counts of cells eluted from lower side of filters after migration versus different chemoattractants. Data are the mean ± SD of 3 different experiments. ** *p* < 0.01, *** *p* < 0.001 compared to unexposed control (CTRL).

Since one of the ways in which GO was known to induce cytotoxicity was through the induction of ROS, we analyzed whether there was an increase of ROS in macrophages after treatment with GO-BSA. As Figure 5A,B showed, there was a surprising decrease of ROS in treated cells. This decrease was concentration dependent and it was extremely clear in THP-1 cells. 

Finally, as a functional test, we studied the chemotaxis of macrophages towards known chemoattractants such as LPS and tumor cell necrotic extracts or GO-BSA, finding, as Figure 5C,D showed, GO-BSA functioned as a chemoattractant for macrophages similarly to necrotic extracts or LPS. 

## 4. Discussion

The present study demonstrated GO nanosheets functionalized with BSA in primary cultures of human leukocytes, derived from peripheral blood, exerted no cytotoxic effect in terms of viability, ROS production, and functionality. These effects are particularly interesting in view of the extensive literature showing the great potential of graphene derived composites as safe drug delivery platforms [31] and reporting on their cytotoxic effects, especially in relation to the immune system [32]. Many studies of graphene cytotoxic effects in tumor cell lines use large amounts of nanomaterials which, in our opinion, are not needed. This is because, according to our data, as much as 125 μg BSA can be uploaded onto 1 μg GO; this can be explained by the high surface/volume ratio of ≈3000 m^2^/g, according to BET measurements (not shown). In the present study, we used two different concentrations of the composites (GO-BSA and GO-BSA-FITC), equivalent to 1.5 μg/mL and 3 μg/mL of GO, in cells from peripheral blood from healthy donors and found both concentrations and times of exposure were safe in terms of viability. In most studies, cell membrane damage in eukaryotic cells was induced by a minimum of 25 μg/mL GO or reduced GO and probably low S/V ratio values, as recently reported [33]. In our previous studies [18,19], we showed low doses of GO obtained from graphite exfoliation were less cytotoxic than the GO obtained by the Hummer-modified method from nanotubes on various cell lines. We have shown here GO had negative zeta potential (−14 mV), due to the number of carboxylic acids that at pH 7.0 were deprotonated (C(=O)O^−^) in aqueous medium. Zeta potential becomes increasingly negative in the composites. In fact, BSA has a low isoelectric point (pH 4.78), and at pH 7.0 it displays negative charges, so when it is bound to FITC and then is adsorbed on GO, it becomes more negative and stably bound. These results are also confirmed by the PDI value, which shows a decreasing trend as the BSA (GO/BSA PDI = 0.453 ± 0.03) and FITC are uploaded on GO (GO/BSA/FITC PDI = 0.382 ± 0.01). According to recent work [34], PDI values < 0.5 indicate a narrow distribution of particles of different sizes, while higher values of PDI show the sample has a much wider distribution range of nanosheet diameters, and is polydispersed. Lower PDI values (<0.1 or <0.2) are recorded in single layer GO dispersions or when GO is highly oxidized [34]. The PDI values recorded in our samples were in line with recent reports on oxidized graphene multilayers [34]. The greater dispersibility of the GO-BSA-FITC composite can be explained by the fact that fluorescein chromophore has a pKa = 10 and the molecule includes a carboxylate group and a phenolic group. The latter, at pH = 7.00, contributes to increased binding on GO nanosheets with hydrophobic and electrostatic interactions or hydrogen bonds and stabilizes the dispersion, which tends towards a monodisperse profile with the lowest PDI (0.382). 

Another crucial factor to be considered in terms of cytotoxicity of nanomaterials is their size, as it determines the uptake by immune cells and may eventually produce an inflammatory response. The report by Ma J. et al. [35] showed GO sheets of smaller lateral size (i.e., 50 nm to 350 nm) tended to be engulfed by macrophages without inducing an inflammatory response, while bigger size nanosheets (beyond and greater than 350 nm) polarizing macrophages into M1 or M2 subsets, was particularly interesting. Conjugation of GO with BSA protein and FITC fluorophore produced better dispersibility in working medium (RPMI) of GO and enabled us to monitor cellular uptake and reduce nanosheet thickness. In addition to the morphological-topographic result, obtained by AFM, the decrease in fluorescence due to quenching confirmed the formation of a stable composite GO-BSA-FITC; it was known GO nanosheets had a great adsorption capacity for proteins like BSA [36] and functionalization with serum proteins improved uptake and biocompatibility [37]. 

Orecchioni et al. [38,39] have shown GO functionalized with amino functional groups did not disrupt cellular homeostasis and induced activation of genes that were crucial for the cross-talk between innate and adaptive response, such as CCL3 and CCL5, or transcription factors of the Th1 response (STAT1) that were central to tumor suppression and elimination [40]. GO functionalization with polyethylene glycol (PEG) has been shown to increase aqueous solubility and, interestingly, to target sensitivity of anti-tumor drugs and even to facilitate cell apoptosis induced by drugs in tumor cells [41]. Another advantage of using GO coated with PEG or BSA was to protect drugs or proteins, delivered into cells as therapeutic agents, from enzymatic cleavage [42]. 

Our objective in this study was to investigate the uptake of the minimum essential dose of nanomaterial able to carry cargo inside cells and to analyze whether the main functionalities of macrophages were affected in the early phases. For this reason, times of exposure to nanomaterials were limited to 24 h. It is known ROS production is the first response of cells to foreign substances and the first step towards cell damage and acute inflammation when unrestricted. However, in our study, GO-BSA nanosheets seem to reduce ROS production in both macrophages and THP-1 cells. An explanation for this decrease might be found in the recent literature on the scavenging activity of free oxygen radicals and the exceptional antioxidative ability of low-dimensional carbon-based nanomaterials [43]. Another aspect investigated in the present study was the ability of GO-BSA to attract macrophages. It was previously reported GO was chemoattractant for *E. Coli* through receptor interactions [44], and although the smallest sized GO had the greatest cellular uptake, it could not induce macrophage polarization and therefore inflammation [45]. The demonstration that small size GO nanosheets were chemotactic for macrophages derived from peripheral blood monocytes and THP-1 cells was reported here for the first time. This implied GO could be a good candidate as an adjuvant for personalized cancer vaccines or as an attractant for tumor associated macrophages (TAMs) from solid tumors with the aim to reprogram them into M1 macrophages. 

## 5. Conclusions

In conclusion, we suggest size and concentration as well as the degree and type of functionalization are determining factors for cytotoxicity. The uploading of GO with BSA produced better adhesion to cell surfaces. And, binding with FITC allowed visualization of nanosheets inside cells. The nanomaterial has been shown not to induce the release of ROS that could trigger an inflammatory response, while at the same time exhibiting chemoattractant properties for macrophages. Future studies should be targeted at 3D culture models with the aim of understanding the penetrative capacity of small-sized GO-NPs, including carbon dots, into solid tumors and their possible use in cancer immunotherapy. 

## Figures and Tables

**Table 1:** Average lateral dimensions and those corresponding to 90% cut-off measurements (in brackets). Equivalent radius represents the radius of a spherical particle having the same value as that of the nanoparticles under consideration.

Composites Size (g)		
Samples	Equivalent Radius in nm Cut Off (90%)	Height in nm Cut Off (90%)
Pristine GO	700 (1500)	340 (770)
GO/BSA	300 (650)	100 (270)
GO/BSA-FITC	115 (185)	8.0 (18)

**Table 2:** Zeta potential/(ξ/mV) and PDI (polydispersity index).

Samples	Zeta Potential (ξ/mV)	Polydispersity Index (PDI)
GO	−14.0 ± 0.12	0.644 ± 0.04
GO/BSA	−38.8 ± 0.14	0.453 ± 0.03
GO/BSA/FITC	−42.8 ± 0.13	0.382 ± 0.01

## Data Availability

The datasets generated and analyzed during the current study are available from the corresponding author upon reasonable request.

## Supplementary Materials

The following supporting information can be downloaded at: https://www.mdpi.com/article/10.3390/biomedicines12020256/s1, Figure S1: Z-potential/(ξ/mV) curves of GO, GO/BSA and GO/BSA/FITC; Table S1: Summary statistics of cell types in Control, GO con and GO samples.
