# Supplementary material for: Biological Effects of Small Sized Graphene Oxide Nanosheets on Human Leukocytes
Source: Biomedicines. 2024 Jan 23;12(2):256. doi: 10.3390/biomedicines12020256 (PMC10887315; doi:10.3390/biomedicines12020256)

Biological effects of small sized graphene oxide nano sheets on human leukocytes

Michele Aventaggiato [1,†], Federica Valentini [2,†], Daniela Caissutti [1], Michela Relucenti [3], Marco Tafani [1], Roberta Misasi [1], Alessandra Zicari [1], Sara Di Martino [1], Sara Virtuoso [4], Anna Neri [5] and Stefania Mardente [1,*]

Supplementary Material

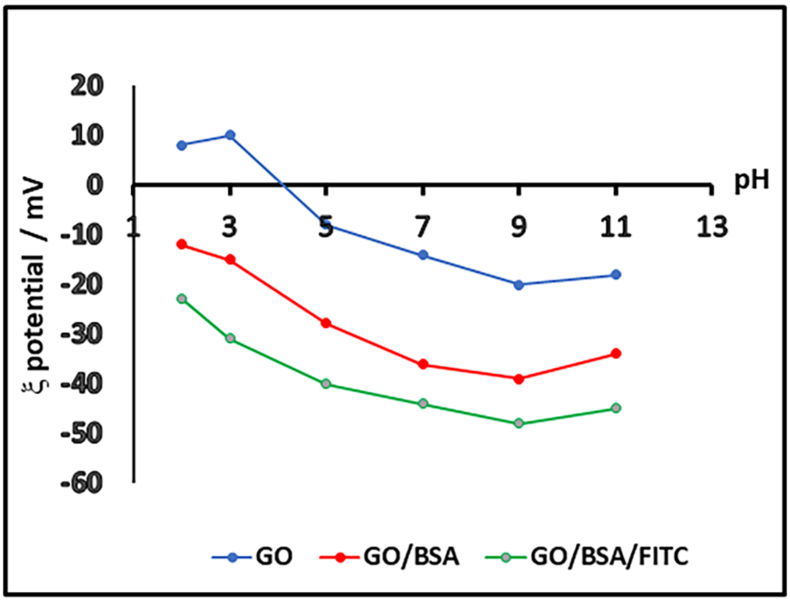

Figure S1. Z-potential/(ξ/mV) curves of GO, GO/BSA and GO/BSA/FITC aqueous dispersions as a function of pH.

Statistical analysis (SEM) -results

Statistical analysis results are summarized in Tab 1. The ratio of circular to elongated shapes is virtually identical in the control and treated samples (about 1.5). This means that most seeded cells adhere to the substrate by taking circular shapes and remain in this morphological arrangement, regardless if they are treated or not.

Table S1. Summary statistics of cell types in Control, GO con and GO samples.

	**Control**			**GO Con**			**Go**		
	Circular	Elongated	Circ/Elon	Circular	Elongated	Circ/Elon	Circular	Elongated	Circ/Elon
**Cell number**	316	216	1,46	293	201	1,45	289	194	1,48
**Mean**	16,63	11,38		15,42	10,57		15,21	10,21	
**Std Error**	1,32	1,06		0,54	0,89		1,03	0,52	
**95% CI**	13,85 to 19,40	9,135 to 13,60		14,27 to 16,57	8,70 to 12,45		13,04 to 17,37	9,10 to 11,31	
	**Tot = 532**			**Tot= 494**			**Tot= 483**		

Hystograms show that according to ANOVA tests there is not a statistically significant difference (F=0.51, P= 0.603) among the different groups of cells (circular, elongated, untreated and treated with the two indicated concentrations of GO-BSA-FITC.

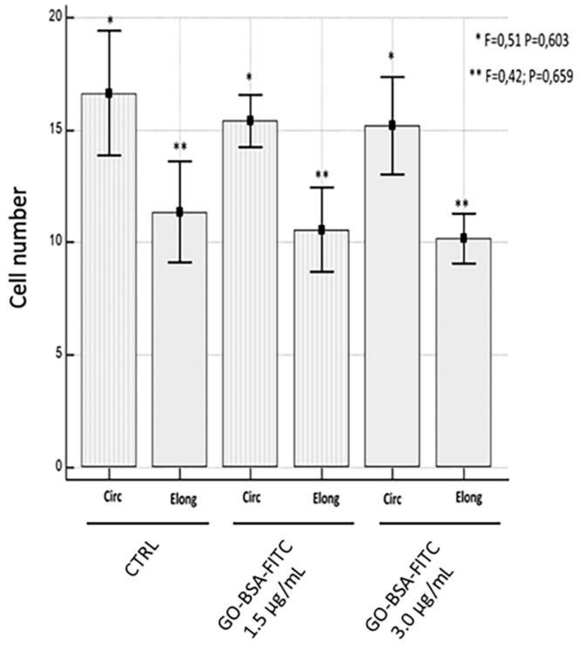

Supplement: Supplementary file 1 [file biomedicines-12-00256-s001.zip › biomedicines-2805786-supplementary.pdf]
